# Natural Antioxidants: Multiple Mechanisms to Protect Skin From Solar Radiation

**DOI:** 10.3389/fphar.2018.00392

**Published:** 2018-04-24

**Authors:** Spencer Dunaway, Rachel Odin, Linli Zhou, Liyuan Ji, Yuhang Zhang, Ana L. Kadekaro

**Affiliations:** ^1^Department of Dermatology, University of Cincinnati College of Medicine, Cincinnati, OH, United States; ^2^Division of Pharmaceutical Sciences, University of Cincinnati College of Pharmacy, Cincinnati, OH, United States

**Keywords:** antioxidants, ultraviolet radiation, oxidative stress, inflammation, skin cancer, reactive oxygen species

## Abstract

Human skin exposed to solar ultraviolet radiation (UVR) results in a dramatic increase in the production of reactive oxygen species (ROS). The sudden increase in ROS shifts the natural balance toward a pro-oxidative state, resulting in oxidative stress. The detrimental effects of oxidative stress occur through multiple mechanisms that involve alterations to proteins and lipids, induction of inflammation, immunosuppression, DNA damage, and activation of signaling pathways that affect gene transcription, cell cycle, proliferation, and apoptosis. All of these alterations promote carcinogenesis and therefore, regulation of ROS levels is critical to the maintenance of normal skin homeostasis. Several botanical products have been found to exhibit potent antioxidant capacity and the ability to counteract UV-induced insults to the skin. These natural products exert their beneficial effects through multiple pathways, including some known to be negatively affected by solar UVR. Aging of the skin is also accelerated by UVR exposure, in particular UVA rays that penetrate deep into the epidermis and the dermis where it causes the degradation of collagen and elastin fibers via oxidative stress and activation of matrix metalloproteinases (MMPs). Because natural compounds are capable of attenuating some of the UV-induced aging effects in the skin, increased attention has been generated in the area of cosmetic sciences. The focus of this review is to cover the most prominent phytoproducts with potential to mitigate the deleterious effects of solar UVR and suitability for use in topical application.

## Impact of solar ultraviolet radiation exposure to human skin

The skin represents the ultimate protection against multiple environmental insults and functions to maintain systemic homeostasis through multiple mechanisms (Slominski et al., 2012). The most ubiquitous of them all is solar UVR, a potent environmental factor that challenges the skin by repetitive exposures. Erythema, edema, sunburn, hyperplasia, premature aging, and the development of nonmelanoma and melanoma skin cancers are part of the array of adverse outcomes associated with solar exposure (Afaq and Mukhtar, 2001). The first line of defense against UVR is the presence of eumelanin, the dark pigment that acts as a scavenger of free radicals, shields genomic DNA and blocks the deeper penetration of UVR in the skin. UVR causes skin damage via two main mechanisms: directly, via absorption of energy by biomolecules and indirectly through increased production of ROS and reactive nitrogen species (RNS). ROS such as superoxide anions, hydrogen peroxide, hydroxyl radicals, singlet oxygen, and lipid peroxyl radicals are byproducts of aerobic life (Davies, 2000). Formation of free radicals in the skin following UVR is well-documented (Baier et al., 2007; Masaki, 2010).

UVR is a potent generator of ROS in the skin and comprises three sub-categories based on the wavelength, UVA (315–400 nm), UVB (280–315 nm), and UVC (190-280 nm). UVA and UVB are the biologically relevant components of solar UVR; the extremely harmful UVC rays are blocked by the stratospheric ozone layer and therefore do not reach the Earth's surface. UVB stimulates the production of superoxide anion radicals through the activation of NADPH oxidase and respiratory chain reactions (Masaki et al., 1995). UVA produces singlet oxygen by an indirect mechanism that involves photosensitizing reactions with internal chromophores such as riboflavin and porphyrin (Masaki et al., 1999), in addition to superoxide anion radical through NADPH oxidase activation (Valencia and Kochevar, 2008). The increased formation of ROS leads to the oxidation and damage of cellular molecules, resulting in altered function (Wondrak et al., 2006).

The carcinogenic effect of UVB is mainly due to its direct absorption by DNA and the generation of UV-signature mutations, cyclobutane pyrimidine dimers (CPDs) and 6-4 pyrimidine pyrimidone dimers (6-4PP) (Cadet et al., 1997). CPDs are the predominant DNA photoproducts, representing about 85% of UVB-induced DNA lesions (de Gruijl et al., 2001). Most of the DNA lesions are repaired by the nucleotide excision repair (NER) pathway (Cleaver, 2000), but if they are not repaired and are present during cell division there is increased chance of DNA mutation (Friedberg et al., 2006).

UVA radiation represents more than 80% of total daily UV exposure and penetrates deep into the skin, damaging DNA and other biomolecules via the production of ROS. Increased levels of ROS cause oxidation of DNA bases, in particular guanine, leading to the formation of lesions such as 8-oxo-deoxyguanine (8-oxodG), a highly mutagenic lesion (Hayakawa et al., 1995). ROS are constantly generated in skin cells but are usually neutralized by a network of non-enzymatic (i.e., glutathione, ascorbic acid) and enzymatic antioxidants. Antioxidant enzymes such as superoxide dismutase (SOD), catalase (CAT), glutathione peroxidase (GPX), glutathione reductase, and thioredoxin reductase (TRX) act in a coordinated manner to keep normal redox homeostasis (Tyrrell, 2012). However, UVA and UVB exposure increases ROS formation to an extent that frequently overwhelms the endogenous antioxidant capacity of the skin (Scharffetter-Kochanek et al., 1997). In addition, UVR causes the depletion of endogenous antioxidants, further enhancing the imbalance that causes oxidative stress (Shindo et al., 1993; Thiele et al., 1998).

**Figure 1:**
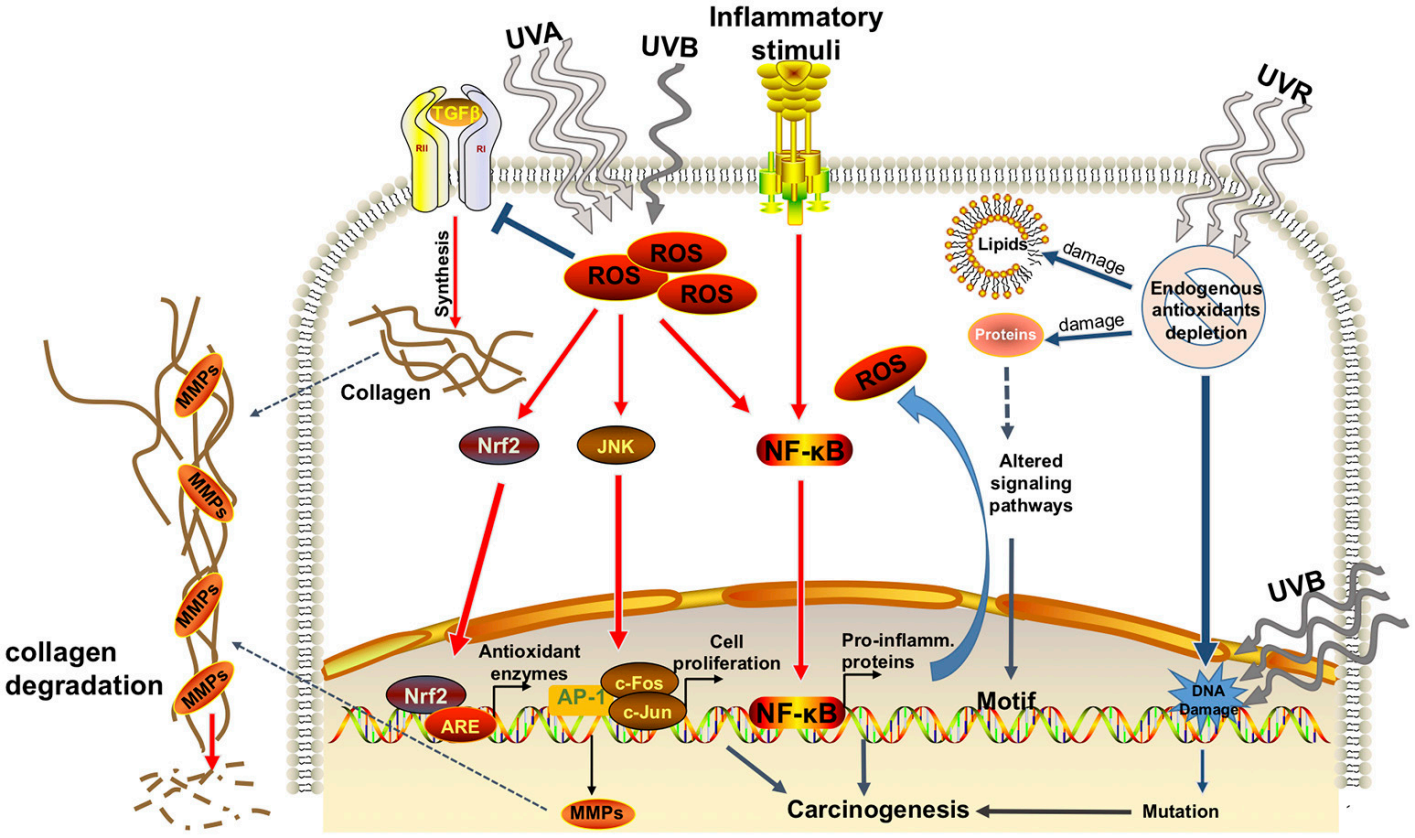
Effects of solar ultraviolet radiation in skin. Simplified representation of the effects of UVR (UVA+UVB) in epidermal keratinocytes. Exposure to UVR induces the formation of reactive oxygen species (ROS). Increase in ROS causes the imbalance between pro-oxidants and anti-oxidants, generating oxidative stress, which in turn, damages lipids, proteins, and DNA. Increases in ROS leads to the imbalance between pro-oxidants and anti-oxidants, generating oxidative stress, which in turn, damages lipids and proteins. Oxidative stress also leads to DNA damage which is compounded by the direct DNA damage known to be produced by UVB. ROS causes the activation of transcription factors such as Nrf2, JNK, and NF-kB. These transcription factors will bind to their specific DNA sequences, antioxidant responsive element (ARE), AP-1 (c-Fos/c-Jun), and NF-kB, respectively. Among the downstream targets of these transcription factors are the phase II antioxidants, and genes associated to promotion of cell proliferation and synthesis of pro-inflammatory mediators (i.e., COX-2, prostaglandin E2, interleukins). Inflammation causes edema, erythema in addition to further increase ROS formation. ROS-induced alteration to lipids and proteins lead to abnormal cellular signaling potentially promoting carcinogenesis. In addition, oxidative stress causes the synthesis and release of matrix metalloproteinases (MMPs) that degrade collagen, a biomarker of skin aging.

In response to excessive presence of ROS a variety of transcription factors are activated including nuclear factor kappa B (NF-kB), activator protein 1 (AP-1) (Cooper and Bowden, 2007), nuclear factor erythroid-derived 2-like 2 (Nrf2), and mitogen-activated protein kinase (MAPK) pathway. The transcription factor Nrf2 is a major transactivator of cytoprotective genes in response to oxidative stress and xenobiotic electrophiles. Nrf2 regulates the transcription of cytoprotective genes by binding to cis-acting elements, the antioxidant response elements (ARE) present in the enhancer regions of these genes (Wasserman and Fahl, 1997; Baird and Dinkova-Kostova, 2011). Activation of NF-kB and AP-1 contribute to the induction of matrix metalloproteinases (MMPs) by dermal fibroblasts that results in extracellular matrix (ECM) protein degradation and premature aging of the skin (Tsuji et al., 2001). Degradation of ECM also facilitate invasion and metastasis of cancer cells (Noël et al., 2008; Figure 1).

UVR upregulates the expression of tumor necrosis factor-alpha (TNF-α), an essential player in the inflammatory response (Li et al., 2013). Inflammation leads to changes in a cascade of cytokines that modulate the immune system, including interleukin-10 (IL-10), IL-4, and prostaglandin E2 (PGE2) (Shreedhar et al., 1998), these molecules in turn, modulate the systemic immune responses through regulatory T-cells. NF-kB activation induces the expression of cyclooxygenase-2 (COX-2) an inflammatory enzyme that converts arachidonic acid into prostaglandins (PGE2) (Mitchell et al., 1995; Figure 1). PGE2 leads to pain and edema as a result of local vasodilation (Cerutti and Trump, 1991). Increased levels of PGE2 are commonly found in squamous and basal cell carcinomas and correlate with their aggressive behavior (Klapan et al., 1992). Elevation of ROS and RNS also causes immunosuppression (Halliday, 2005) and UV-induced systemic immunosuppression has been demonstrated in animal models. Antigen-specific regulatory T-cells (CD4+CD25+foxp3+ cells) produced by irradiated animals could be transferred to non-irradiated mice which then became immune tolerant (Ghoreishi and Dutz, 2006). Skin mast cells are important players on the mechanism of UV-induced systemic immunosuppression. It was demonstrated that the number of mast cells migrating from the skin to draining lymph nodes correlated with the degree of UV-induced immunosuppression (Byrne et al., 2008). In addition, regulatory B-cells contribute to UV-induced immune suppression by modulating dendritic cell-mediated T-cell activation (Byrne and Halliday, 2005). Under normal circumstances the emergence of malignant cells is readily identified by immune-surveillance mechanisms. However, UVR induced immunosuppression compromises this ability and facilitates the development of skin tumors.

Presence of extensive amounts of DNA damage can block RNA transcription leading to the activation of p53 and induction of apoptosis (Batista et al., 2009) and, consequent removal of potentially mutated cells. However, DNA replication in the presence of UVB-induced photo-lesions increases the formation of transition mutations (C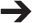 T) and double mutations (CC
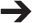
TT) at adjacent pyrimidine sites (Brash et al., 1991; De Gruijl and Rebel, 2008) as well as, T
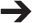
G transversions from the oxidative intermediate 8-oxodG (Kino and Sugiyama, 2005). In addition, ROS-induced by UVA causes oxidation of protein and lipids, altering normal cellular signaling that may promote abnormal cell proliferation. For example, indirect photo-oxidation of proteins caused by oxygen radicals and hydroxyperoxides reacting with amino acid residues can cause changes to protein conformation by misfolding and deeply affect the protein functionality (Pattison et al., 2012; Venza et al., 2015; Figure 1).

Exposure to UVR has been reported to induce epigenetic changes; which may contribute to the development of skin cancer. Epigenetic changes include DNA methylation, histone modifications, and miRNA-mediated gene regulation (Katiyar et al., 2012). DNA methylation regulates gene expression by adding a methyl group to the 5th carbon of cytosine in a CpG dinucleotide. Analysis of human methylation profile revealed that the distribution of CpG dinucleotides is not random but rather, clusters form in certain portions of the DNA. These CpG islands are often located upstream to the promoter region of over half of the human genes (Takai and Jones, 2003). It is not surprising therefore that studies revealed a distinct landscape of DNA methylation in cancer cells in contrast to the one displayed by normal cells. One example is the high incidence of methylation found in critical genes of melanoma samples, specifically the hypermethylation of CDKN2A that silences the expression of protein p16 (Baylin and Ohm, 2006). Recent progress in epigenetic studies revealed that loss of the DNA hydroxymethylation mark, 5-hydroxymethylcytosine and high levels of DNA methylation in the promoter region of several tumor suppressor genes are observed in melanoma (Fu et al., 2017).

## Natural products and skin photo-protection

Increasing incidence of skin disorders has resulted in high demand for the research community to explore and develop superior alternatives for skin photo-protection. Traditional sunscreens contain inorganic compounds such as titanium dioxide (TiO2) and zinc oxide (ZnO), minerals that reflect and scatter UVA and UVB rays. However, these inert particles are often visible as an undesirable opaque layer on the skin. In addition, increased concern has been raised about the safety of these metal-based particles and their potential toxicity (Smijs and Pavel, 2011). Recognition of the detrimental role of UVA has led to extensive research exploiting the active ingredients of natural products. Antioxidants have the potential to enhance the endogenous capacity of the skin and help neutralize ROS-induced by external factors such as solar UV radiation.

Nearly all life on earth developed mechanisms to combat the effects of UVR. Skin developed many photo-adaptive mechanisms including the production of antioxidants and UV-absorbing metabolites as part of an auto-preserving response (Saewan and Jimtaisong, 2015). For this reason, investigators have examined cutaneous compounds in search of products with UV protecting properties. For example, Vitamin D and its metabolites have been found to improve keratinocyte survival after UVB irradiation (Gupta et al., 2007), enhancing DNA repair and decreasing CPD production (Gordon-Thomson et al., 2012; Slominski et al., 2015). Indeed, studying cutaneous compounds has a great potential for improvements in therapy. However, investigators should not limit themselves to the skin; plants also have developed unique and effective mechanisms of dealing with UVR. In the last decade, there has been a worldwide trend toward the development of products containing organic compounds. Plants offer a source of UV protecting compounds that can be used to substitute or reduce the quantity of synthetic agents in cosmetic products.

Botanical compounds belonging to multiple chemical classes including polyphenols, monoterpenes, flavonoids, organosulfides, and indoles have been shown to have anti-mutagenic and anti-carcinogenic properties, without causing significant toxicity when administered topically or orally in mouse models. The mode of action of these compounds includes stimulation of anti-inflammatory and immune responses, detoxification, modulation of antioxidants, and alteration of gene expression (Saewan and Jimtaisong, 2015). Continuing investigation has revealed that these compounds act through several pathways and therefore, maintain tissue homeostasis through multiple mechanisms (F'Guyer et al., 2003). In this review, we discuss some of the innumerous published studies evaluating the properties and beneficial effects of botanical natural compounds, with particular emphasis on skin photo-protection (Table 1). We also discuss some of the known mechanisms associated to the protective effects of these phytoproducts. Due to the size constraints of this article, several studies were not mentioned, we apologize to the authors not included in this review.

**Table 1:** Multiple mechanisms of photoprotection by natural phytoproducts.

**Antioxidant**		**Anti–lnflammatory**			**Direct UVR Blocking**	**MMP/Collagen**	
**Antioxidant (General)**	**Nrf2/Keap1**	**Non–specified**	**MAPK/JNK/AP1**	**NFkB/TNF/COX2**		**Wnt/EMT**	**DNA**
**Tea Polyphenols**	**Grape Polyphenols**	**Tea Polyphenols**	**Tea Polyphenols**	**Tea Polyphenols**	**Algae**	**Grape Polyphenols**	**Tea Polyphenols**
Agarwal et al., 1993 Katiyar et al., 2001 Vayalil et al., 2004 Katiyar, 2002 **Grape Polyphenols** Afaq et al., 2003a Lephart et al., 2014 Liu et al., 2011 **Honokiol** Dikalov et al., 2008 **Quercetin** Seo et al., 2015 Ma et al., 2015 Chen et al., 2017 **Apocynin** Stolk et al., 1994 **Aloe Vera** Imaga et al., 2014 Rodrigues et al., 2016 **Silymarin** Radek et al., 2007 **Algae** Heo et al., 2009 Hyun et al., 2013 **Propolis** Kim and Yoo, 2016 Batista et al., 2018 Bolfa et al., 2013 **Melatonin** Esferoglu et al., 2005 Pérez-González et al., 2017 Janjetovic et al., 2014	Liu et al., 2011 **Sulforaphane** Thimmulappa et al., 2002 Talalay et al., 2007 Kubo et al., 2017 **Melatonin** Janjetovic et al., 2017	Agarwal et al., 1993 **Honokiol** Vaid et al., 2010 Costa et al., 2017 **Quercetin** Seo et al., 2015 Ma et al., 2015 **Aloe Vera** Bałan et al., 2014 **Tumeric** Sandur et al., 2007 **Silymarin** Sharifi et al., 2012 Oryan et al., 2012 **Propolis** Cole et al., 2010 **Melatonin** Sierra et al., 2013 Scheuer et al., 2016	Nomura et al., 2000 Katiyar et al., 2001 **Grape Polyphenols** Lephart et al., 2014 Afaq et al., 2003a Lee and Moon, 2006**Apocynin** Byun et al., 2016**Tumeric** Guo et al., 2008	Afaq et al., 2003b Agarwal et al., 1993 **Grape Polyphenols** Lee and Moon, 2006 Afaq et al., 2003a**Quercetin** Chen et al., 2017 **Honokiol** Prasad et al., 2017 Vaid et al., 2010 **Apocynin** Byun et al., 2016 Nam et al., 2016 **Tumeric** Guo et al., 2008 Jang et al., 2012 **Melatonin** Carrillo-Vico et al., 2005 Esposito and Cuzzocrea, 2010 Lee et al., 2016 **Ginseng** Lee et al., 2012	Yang et al., 2018 Hartmann et al., 2016 **Propolis** Gregoris et al., 2011	Lephart and Andrus, 2017 Lee and Moon, 2006 **Honokiol** Zhao et al., 2017 **Aloe Vera** Misawa et al., 2017 **Tumeric** Jang et al., 2012 **Algae** Ryu et al., 2009 Urikura et al., 2011 **Tumeric** Liang et al., 2017	Michna et al., 2003 **Grape Polyphenols** Cao et al., 2009 **Silymarin** Katiyar et al., 2011 Guillermo-Lagae et al., 2015 **Algae** Heo and Jeon, 2009 **Melatonin** Jang et al., 2012; Janjetovic et al., 2017 **Honokiol** Prasad et al., 2017

## Melatonin

Melatonin was first described as a neuroendocrine product of the pineal gland (Lerner et al., 1958) and is known to drive the circadian rhythm in vertebrates, including humans (Hardeland et al., 2011). Endogenous melatonin is synthesized from tryptophan via 5-hydroxytryptamine (Slominski et al., 2002; Acuña-Castroviejo et al., 2014) and is produced in several tissues types, including the skin (Slominski et al., 1996; Andrzej Slominski et al., 2002). Further, melatonin receptors are found in the skin, suggesting that it is a potential target for the indoleamine (Slominski et al., 2005). The details of the cutaneous serotonergic/melatoninergic system are outside the scope of this review and has been thoroughly discussed in other reviews (Slominski et al., 2005). Melatonin is found in phylogenetically ancient organisms like cyanobacteria and algae (Hardeland et al., 1995) and in a wide variety of terrestrial plants including nuts, beans, mushrooms, and Scutellaria biacalensis, a traditional Chinese herbal medicine (Hardeland, 2016; Meng et al., 2017). Melatonin has long been recognized as a potent scavenger of ROS, exhibiting antioxidant effects that prevent cell damage and inflammation (Acuña-Castroviejo et al., 2014). Recent work has extensively discussed the photo-protective and antioxidant properties of melatonin (Slominski et al., 2017, 2018). Herein, we will briefly highlight some of the aspects most relevant for this review. Melatonin has been shown to protect against UV radiation (Fischer et al., 2008). Pinealectomized (i.e., melatonin-deficient) rats were reported to show increased lipid peroxidation and decreased expression of antioxidant enzymes such as catalase and glutathione peroxidase. Replacement of melatonin in these animals reverted this effect (Esferoglu et al., 2005). Melatonin was shown to be a photoprotective agent via modulation of proinflammatory mediators (Carrillo-Vico et al., 2005; Esposito and Cuzzocrea, 2010). One study conducted *in vivo* using Wistar rats evaluated the benefits of melatonin in topical sunscreen emulsions. The results demonstrated that melatonin protected against UV-induced erythema as well as activated endogenous enzymatic protection against oxidative stress (Sierra et al., 2013). Studies have demonstrated a decrease in the production of ROS after UVB exposure in both human melanocytes (Janjetovic et al., 2017) and keratinocytes treated with melatonin (Janjetovic et al., 2014). This effect was accompanied by enhanced p53 expression, improved DNA repair and decreased CPD generation. While the mechanisms underlying these effects are not fully understood, it appears that at least in part, melatonin and its metabolites exert their effects through activation of Nrf2. Silencing of Nrf2 in melanocytes resulted in reversal of the protective effects of melatonin (Janjetovic et al., 2017). Sirt1, a member of the sirtuin family of deacetylases, was found to be critical to melatonin's anti-oxidative properties, since silencing of SIRT1 in keratinocytes, reversed the protection against peroxide-induced damage and cell death (Lee et al., 2016). The list of Sirt1 substrates is continuously growing and include several transcription factors, including the tumor suppressor p53, members of the FoxO family, peroxisome proliferator-activated receptor gamma, and NF-kB (Rahman and Islam, 2011). Some of the metabolites of melatonin, such as 4-hydroxymelatonin, have been shown to be even stronger antioxidants than melatonin itself (Pérez-González et al., 2017). A recent randomized, placebo-controlled clinical study was conducted to evaluate the sun protective effect of topically applied melatonin. The results of this study revealed a significant dose-dependent change in erythema formation between treated and non-treated areas (Scheuer et al., 2016). These results are encouraging and further studies exploring the potential use of melatonin and its metabolites compounded into topical applications could lead to the development of new dermatologic treatments.

## Tea polyphenols

Several studies have reported the antioxidant and anti-inflammatory properties of polyphenols. Polyphenolic compounds have been extensively studied and are found in several plants including tea leaves, grape seeds (*Vitis vinifera*), blueberries (*Vaccinium myrtillus;* Svobodova et al., 2008), almond seeds (*Prunus amygdalus;* Wijeratne et al., 2006), and pomegranate extract (*Punica granatum;* Afaq et al., 2009). The beneficial properties of polyphenols have been supported by several studies performed in skin cells, skin reconstructs, and human skin; for this reason these compounds have been increasingly incorporated into cosmetic and medicinal products (Nichols and Katiyar, 2010; Ndiaye et al., 2011). Polyphenols found in tea leaves and grape extracts have been studied the most extensively and are addressed individually in the following sections.

Freshly harvested tea leaves can be processed in different ways to generate oolong tea, green tea or black tea; with each subtype containing different properties (Graham, 1992). The main polyphenols present in green tea are the catechins gallocatechin (Mukhtar et al., 1992), epigallocatechin (EGC), and epigallocatechin-3-gallate (EGCG). Studies have shown that EGCG inhibits UVB-induced release of hydrogen peroxide from cultured normal epidermal keratinocytes and suppresses the phosphorylation of the MAPK (Katiyar et al., 2001). In addition, EGCG reduces inflammation through the activation of NFkB (Afaq et al., 2003b). Green tea also contains other phenolic acids such as gallic acids and theanine as well as the alkaloids caffeine, theophylline, and theobromine (Katiyar et al., 2000). Theaflavins, present in black tea, have been found to inhibit the UVB-induction of AP-1, suppressing the extracellular-regulated kinase (ERK) and c-jun N-terminal kinase (JNK) (Nomura et al., 2000). Tea polyphenols are capable also of blocking the UVB-induced activation of phosphatidyl-inositol 3-kinase (IP3K) (Nomura et al., 2001).

In animal models topical or oral administration of tea polyphenols showed protection against UVB-induced carcinogenesis and inflammation. Long-term feeding of SKH-1 hairless mice with tea polyphenols followed by UVB irradiation resulted in blockade of edema, counteraction of antioxidant depletion and abrogation of inflammation marker cyclooxygenase-2 (COX-2) expression (Agarwal et al., 1993). At the molecular level, oral administration of green tea to SKH-1 mice enhanced the number of UV-induced p53- and p21-positive cells as well as apoptotic sunburn cells (Michna et al., 2003). Another mechanism by which tea polyphenols exert photo-protection is by counteracting UVB-induced local and systemic immunosuppression, besides decreasing ROS in the skin (Katiyar et al., 1999). EGCG counteracts UVR induced alterations in the IL-10/IL-12 cytokines. This is possibly mediated through antigen-presenting cells in the skin and draining lymph nodes or by blocking the infiltration of IL-10 secreting CD11b+ macrophages into the irradiated site. EGCG also inhibits the migration and depletion of antigen presenting cells (APCs) and significantly decreases dermal and epidermal hydrogen peroxide and nitric oxide production (Katiyar, 2002; Vayalil et al., 2004).

## Grape seed polyphenols

Grape seeds are very rich in polyphenols, among them catechin, epicatechin, and oligomeric proanthocyanidins. Many of the beneficial effects of grape seeds such as its antioxidant, anti-inflammatory and anti-proliferative activity come from the polyphenolic phytoalexin component resveratrol. Resveratrol (3,5,4′-trihydroxy-trans-stilbene) has been the most extensively studied polyphenol. Its beneficial properties were first described in 1997 (Jang et al., 1997), which was shortly followed by an increasing number of studies supporting the initial findings (Zhao et al., 1999). The protective benefits of resveratrol were demonstrated *in vivo* studies showing significant inhibition of UV-mediated edema and inflammation with topical application onto SKH-1 hairless mice prior to UVB exposure. At the molecular levels it was found that the protective effects of resveratrol occurred through inhibition of the inflammation mediator COX-2, inhibition of ornithine decarboxylase (ODC), reduction of hydrogen peroxide and decreased lipid peroxidation (Afaq et al., 2003a). The properties of resveratrol as an anti-oxidant, anti-cancer and anti-inflammatory agent are most likely due to changes in gene expression (Lephart et al., 2014). Resveratrol significantly increased the production of antioxidants such as catalase and superoxide dismutase (SOD) as well as expression of SIRT1 and extracellular matrix (ECM) proteins such as collagen and elastin. In addition it significantly inhibited pro-inflammatory mediators (IL-1A, IL-6, IL-8) and biomarkers of skin aging, such as transforming growth factor beta 1 (TGFB1), transforming growth factor beta receptor 2 (TGFBR2), tumor necrosis factor receptor 1A (TNFRSF1) and S100 calcium-binding protein A8 (S100 A8) (Lephart and Andrus, 2017). The antioxidant property is an essential aspect of the protective effect of resveratrol. Studies have revealed that resveratrol attenuates UVA-induced oxidative stress in human keratinocytes by downregulating Keap1, a protein that binds to Nrf2 and tags the antioxidant transcription factor to degradation (Liu et al., 2011). In addition, SIRT1 confers protection against UVB and ROS-induced cell death by modulating p53 and c-Jun N-terminal kinase (JNK) (Cao et al., 2009). Resveratrol was found to inhibit tumor necrosis factor (TNF)-alpha-induced proliferation and matrix metalloproteinase (MMP) expression through the inhibition of nuclear factor-kappa B (NFkB) and activator protein-1 (AP-1) in smooth muscle cells (Lee and Moon, 2006). Whether or not these mechanisms could be also observed in skin needs to be verified. In summary, studies have shown that resveratrol exerts its beneficial effects through multiple pathways, including changes in gene expression which ultimately results in overall improvement of skin health.

Resveratrol application in commercial products still represents a challenge due to its rapid metabolism, which is particularly fast at the systemic level (Baur and Sinclair, 2006). For this reason, resveratrol seems to be more suitable for topical use. *In vitro* studies using Raman spectroscopy and analysis of progressive tape stripping of the stratum corneum revealed that topically applied resveratrol penetrates deep into the viable upper layers of the epidermis. The percentage of resveratrol found in the stratum corneum after permeation was however very low, about 5% of the total applied, but still enough to cause a significant impact on the epidermal antioxidant capacity (Alonso et al., 2017). Advances in the nano-delivery technology represents a great progress toward enhancement of skin retention and bioavailability of compounds with poor permeation capacity. The use of resveratrol encapsulated in lipid nanoparticles resulted in greater skin uptake and consequently, increased protection, and antioxidant activity (Gokce et al., 2012). To improve its efficacy for cosmetic and medical applications, multiple studies have been conducted to develop resveratrol analogs that are more stable and more biologically active than the natural form. There is evidence that some of the analogs have great potential to be used for topical applications (Sirerol et al., 2015), especially considering that some were found to display increased potency and longer half-life compared to resveratrol (Lephart and Andrus, 2017). It is important that future studies determine how chemical modifications to resveratrol impact its beneficial effects in topical application and perhaps, even its bioavailability after oral ingestion.

## Honokiol (*Magnolia sp*)

Honokiol is a small-molecule, a hydroxylated biphenolic compound isolated from *Magnolia officinalis* and other plants of the *Magnolia* genus. Honokiol has been used in traditional Chinese medicine for thousands of years. Recently western medicine has begun to recognize its multiple pharmacological properties as an antioxidant (Dikalov et al., 2008), anti-inflammatory (Vaid et al., 2010), antiangiogenic (Fried and Arbiser, 2009), anticarcinogenic (Leeman-Neill et al., 2010; Mannal et al., 2011), and antibacterial (Park et al., 2004). Honokiol is known to reduce inflammation caused by ROS and was shown to protect HaCat keratinocytes and human fibroblasts against the damaging effects of cigarette smoke by reducing the inflammatory mediator IL1-α and degradation of collagen (Costa et al., 2017). The anti-inflammatory role was also shown to protect against UVR; in a mouse model honokiol significantly decreased UVB induced expression of inflammatory mediators including COX2, IL-1, and IL-6 (Vaid et al., 2010). Honokiol also modulates the expression of cell cycle proteins in melanoma, resulting an anti-neoplastic effects (Guillermo-Lagae et al., 2017). Induction of immunosuppression by UVR plays a fundamental role in the development of cutaneous carcinogenesis. Recently, topical application of honokiol was found to reverse UV-induced immunosuppression, significantly decreasing the suppression of contact sensitivity in CeH/HeN mice. This effect was shown to be mediated through inhibition of COX-2 expression and subsequent PGE2 production. Interestingly, the study also found evidence that honokiol inhibits UVB-induced DNA hyper-methylation (Prasad et al., 2017). Because DNA hyper-methylation is known to affect numerous genes involved in the initiation or promotion of cancer, this newly described attribute of honokiol suggests that this natural antioxidant has a great potential to be employed in photo-protection products.

## Quercetin

Quercetin is a flavonoid, a polyphenolic compound commonly found in plant pigments. Numerous studies have shown that quercetin exhibits several beneficial biological properties including antioxidant, anti-inflammatory (Seo et al., 2015), cardio-protective (Larson et al., 2012), and anti-carcinogenic (Kiekow et al., 2016). Quercetins strong antioxidant capacity comes from several hydroxyl groups present in its structure. Quercetin was shown to be effective in attenuating psoriatic lesions in an imiquimod-induced psoriasis-like mouse model. It was demonstrated that this effect occurred through reduction in the serum levels of TNF-α, IL-6 and IL-17 and increased activation of GSH, catalase and superoxide dismutase. Down-regulation of NFkB, IKKa, NIK, and RelB expression (Chen et al., 2017) were associated with these effects. The properties of quercetin give it great potential to prevent UVR-induced skin damage; however, its poor stability, permeability and solubility are a major drawback for its use in topical application products (Hung et al., 2012). More adequate carriers for quercetin and nanoparticles prepared with biodegradable polymers have emerged as a promising percutaneous delivery system. Formulation of nanoparticles with polymers such as poly (D,L-lactide-co-glycolide) (PLGA) has been shown to improve quercetin topical delivery to mice skin, leading to effective attenuation of the UV-induced damage (Zhu et al., 2016). Liposome nanoparticles have also been studied and they seem to be of great value in skin delivery. It is suggested that liposomes adhere to the skin's surface and subsequently mix with the lipid matrix of the stratum corneum, increasing the partitioning of agents. An *in vivo* model of wound healing in which quercetin and resveratrol were incorporated into oleic acid-based liposomes showed significant reduction in ROS and the counteraction of the inflammatory responses provoked by TPA. The data revealed an improved delivery of the polyphenols, resulting in a faster tissue repair and wound closure (Caddeo et al., 2016).

## Sulforaphane

The natural antioxidant sulforaphane, found in broccoli, exerts anticarcinogenic, antidiabetic and antimicrobial properties. Sulforaphane extracts applied topically onto mouse skin protected against UVR-induced inflammation and edema through the activation of Nrf2 and consequent up-regulation of phase 2 antioxidant enzymes (Thimmulappa et al., 2002). Studies have shown that during the aging process the activity of Nrf2 declines. The causes for the diminished activity of Nrf2 is not understood however, there is evidence that Nrf2 loses the ability to bind to the antioxidant response element (ARE) sequence in antioxidant genes (Suh et al., 2004). Importantly, the ability of Nrf2 to bind to the cis-element is apparently reversible by Nrf2 agonists such as α-lipoic acid (Suh et al., 2004) and sulforaphane (Kubo et al., 2017). Sulforaphane was shown to restore the transactivation ability of Nrf2 and to provide cytoprotection against UVB-induced injury of human lens epithelial cells not only by enhancing the expression of phase 2 enzymes, but also by increasing the antioxidant enzyme catalase (Kubo et al., 2017). The restoration of the Nrf2 activity in aging cells, as well as cells exposed to UVB provides the proof-of-concept that sulforaphane is a natural compound with important preventative and therapeutic effects.

## Apocynin

ROS are produced by mitochondria, peroxisomes, cytochrome P450, and also by NADPH oxidase (Bedard and Krause, 2007). Apocynin is a vanilloid compound naturally found and obtained from the root extract of the medicinal herb *Picrorhiza kurroa*. Apocynin is a known inhibitor of NADPH oxidase activity and exerts its activity by blocking the formation of the NADPH oxidase complex (Stolk et al., 1994). Apocynin is commonly used as a standard NOX inhibitor and it has been shown that it undergoes dimerization in a peroxidase-mediated manner. The resulting homodimers display greater antioxidant capacity than apocynin in monomeric form (Stefanska and Pawliczak, 2008). The therapeutic applications of apocynin have been explored in various health conditions including asthma, neurodegenerative disease, cardiovascular diseases, arthritis (Narayanan et al., 2010), inflammation (Nam et al., 2014), and cancer (Jantaree et al., 2017). As is the case in skin cancer, ROS are a well-known contributor to the pathogenesis of prostate cancer. It was found that apocynin inhibited the production of ROS and reduced the formation of oxidative DNA damage in a rat model of prostate carcinogenesis. These effects were associated with the inactivation the MEK-ERK1/2 pathway, downregulation of cyclin D1 and G0/G1 call cycle arrest (Suzuki et al., 2013). Recently it was shown that apocynin has a strong inhibitory effect on UVB-induced carcinogenic signaling, suggesting a therapeutic potential against inflammation and skin carcinogenesis. In the study, apocynin significantly decreased the UVB-induced activation of AP-1 and NF-kB in JB6 P+ cells and suppressed the promoter activity of UVB-induced COX-2. These effects were then confirmed *in vivo* using topical application of apocynin in a two-stage mouse model. The results showed that apocynin delayed the onset of UVB-induced skin tumors as well as significantly reduced the total number of tumors (Byun et al., 2016). Apocynin was also demonstrated to attenuate the production of inflammatory mediators in keratinocytes, including the Akt, mTOR and NF-kB pathways (Nam et al., 2016). Apocynin has produced therapeutic effects in many disease models and taken together, the multiple activities of this NOX2 inhibitor demonstrate the need for further exploration of its use in skin photo-protection.

## Aloe vera

Aloe vera leaf extracts contain several active components such as proteins, minerals, carbohydrates, and vitamins. Aloe vera demonstrates several beneficial properties including antioxidant (Imaga et al., 2014), antibacterial (Ali et al., 2014), anti-inflammatory and as immunity regulator (Bałan et al., 2014). The antibacterial properties of Aloe vera gel has a great potential to be used for the treatment of skin conditions such as acne vulgaris (Hajheydari et al., 2014). In HaCaT keratinocytes, aloe vera was shown to decrease UVA induced redox imbalance, decrease UVA associated lipid membrane oxidation and increase overall cell survival (Rodrigues et al., 2016). In a mouse model, oral supplementation with aloe vera led to a reduction in UVB induced apoptosis of epithelial cells and a decrease in MMP-2 and MMP-13 formation as well as decreased depth of UV associated wrinkling (Misawa et al., 2017). In addition, studies exploring the effects of combination of natural antioxidants for skin topical application has been showing promising results. Encapsulation of aloe vera and curcumin for topical delivery, resulted in enhanced antioxidant protection (Kitture et al., 2015). The benefits of different combinations of phytoproducts has just began to be studied and represent a great area to be explored.

## Turmeric (curcumin)

Turmeric is a commonly used spice that was shown to have anti-inflammatory properties (Sandur et al., 2007). The active components of turmeric are bisdemethoxycurcumin, demethoxycurcumin and curcumin (Jurenka, 2009). Curcumins exert their anti-inflammatory effects by the inhibition of NFkB and MAPK signaling pathways with reduction of the expression of inducible nitric oxide (iNOS) and COX2 (Guo et al., 2008). Curcumins also inhibit UVB-induced TNF-α at the mRNA level and reduce the expression of matrix metalloproteinase-1 (MMP-1) expression in keratinocytes and fibroblasts (Jang et al., 2012). A recent study demonstrated that tobacco smoke, an important risk factor for skin cancer, induced epithelial-mesenchymal transition (EMT) through Wnt/b-catenin pathway and that curcumin reversed the effect (Liang et al., 2017). Curcumin anticancer activity seems to occur through the inhibition of Sonic hedgehog and Wnt/b-catenin pathway resulting in reduction in the expression of cancer stem cell markers such as CD44, ALDH1A1, Nanog, and Oct 4, demonstrated in breast cancer cells (Li et al., 2018) and in lung cancer cells (Zhu et al., 2017). Whether or not these effects could be observed in skin cancer cells has not been addressed and deserves to investigation.

## Silymarin milk thistle (*Silybum marianum*)

The main chemical components of silymarin that exhibit antioxidant properties are silibinin A and silibinin B (Radek et al., 2007). *In vivo* topical application of silymarin in rats undergoing wound healing led to increased epithelization and decreased inflammation (Sharifi et al., 2012). There is evidence that silibinin accelerates wound healing by increasing the levels of stromelysine 1 (STM1) gene and ECM constituents including glycosaminoglycans and collagen (Tabandeh et al., 2013). It was also reported that silymarin is able to enhance the repair of UVB-induced DNA damage through the NER pathway (Katiyar et al., 2011). It was found that treatment of UVB-irradiated human dermal fibroblasts with silibinin resulted in accelerated DNA repair due to increased XPA and GADD45α mRNA transcripts, in a mechanism dependent on p53 (Guillermo-Lagae et al., 2015). Altogether, these results support the use of silymarin as a potential effective and non-toxic agent for treatment of a diversity of skin inflammatory conditions and in skin cancer prevention.

## Ginseng (*Panax ginseng*)

Ginseng has a place in the global market as a health food and has been used as an active ingredient in herbal cosmetics. The major active components of ginseng are ginsenosides, a diverse group of steroidal saponins that promote ginseng's ability to target a large number of tissues. Ginseng has other active ingredients such as phenolic compounds, polysaccharides, and proteins (Jin et al., 2015). Ginseng is known to exert anti-inflammatory activity by reducing nitric oxide production and iNOS mRNA synthesis in HaCaT keratinocytes and human dermal fibroblasts (Lee et al., 2012). Ginseng was also reported to inhibit the UVB-induced COX2 expression and TNF-α transcription in HaCaT keratinocyte cells (Lee et al., 2012). Recently, ginseng extract was found to attenuate atopic dermatitis (AD), a chronic and relapsing inflammatory skin disease. It is known that the mammalian target of rapamycin (mTOR)/p70 ribosomal protein S6 kinase (p70S6K) signaling is activated in AD. Ginseng extract was found to decrease the phosphorylation of p70S6K (Osada-Oka et al., 2017).

## Algae

In recent years several studies have demonstrated the photo-protective effects of different groups of multicellular algae. Mycosporine like-like amino acids (MMAs) are strong UV absorbing compounds that are abundantly produced by many species of algae and have been incorporated into commercial sunscreens for many years (Hartmann et al., 2016; Yang et al., 2018). In addition to UV absorbing properties, algae extract also can protect against UVR induced ROS. Methanol extraction of a red algae, Corallina pilulifera, showed potent antioxidant activity, protecting against UVA radiation-induced oxidative stress. At the molecular level, it was found that the extract reduced the expression of the matrix metalloproteinases MMP-2 and MMP-9 (Ryu et al., 2009). Many species of brown algae have shown photoprotective effects. Ecklonia cava is rich in polyphenols that are effective at protecting against photo-oxidative stress (Heo et al., 2009). Similarly, extract from Unidaria crenata has been shown to have significant free radical scavenging capabilities and decreased UVB induced apoptosis as well as lipid and protein oxidation in keratinocytes (Hyun et al., 2013). A carotenoid named fucoxanthin, isolated from the brown algae Sargassum siliquastrum was shown to have the property to reduce fibroblast apoptosis caused by UVB exposure (Heo and Jeon, 2009). Fucoxanthin is found in many other species of brown algae including Undaria, HIjikia and Sargassum; it has been shown to decrease UVB induced photoaging in mice by reducing VEGF and MMP-13 expression (Urikura et al., 2011). Other components from the brown algae sargassum sagamianum, including plastoquinones, sargaquinoic acid, and sargachromenol, have been shown to exert protection against UVB; further demonstrating the abundant potential of photoprotective compounds existing within algae extract (Hur et al., 2008).

## Propolis

Propolis is a plant resin collected by honeybees. Its chemical composition and biological activity is very complex and depends on the sources of plants visited by the honeybees (Greenaway et al., 1990; Marcucci, 1995). Propolis is known to offer photoprotection against UVR at and can be compounded to have an SPF level on par with many commercial agents (Gregoris et al., 2011). In addition to its UV absorbing properties, propolis's anti-inflammatory (Cole et al., 2010), and antioxidant abilities make it an attractive natural dermatologic product. Propolis has been shown to significantly reduce UVA induced ROS production in HaCaT cells and to protect against UV induced apoptosis (Kim and Yoo, 2016). Several studies have confirmed the photoprotective and anti-oxidant effect of propolis *in vivo* using both mouse and rat models (Bolfa et al., 2013; Batista et al., 2018) The anti-inflammatory effects of propolis have also been demonstrated as an effective mechanism of promoting wound healing. In one study propolis and other honey products were loaded into chitosan nanofibers and tested against multidrug-resistant Pseudomonas aeruginosa. Results demonstrated great biocompatibility, potent antibacterial activity and enhanced wound-healing properties, suggesting their potential use in wound-healing dressings (Sarhan and Azzazy, 2017). Propolis extracts contain a wide variety of chemical components including a high content of polyphenols, especially flavonoids which include quercetin, pinocembrin, formononetin, and coumaric acid. Further, the composition of extracts can vary dramatically and are known to differ in their antioxidant properties based on the area of origin (Fonseca et al., 2011). It is still unclear which of the many components contribute to propolis's unique properties, making it an excellent source for new research.

## Conclusion and future challenges

Solar ultraviolet radiation in the form of UVA and UVB induce tissue damage in part via the production of ROS. The increase in ROS results in skin inflammation, characterized by vasodilatation that leads to formation of edema and erythema. This process leads to local accumulation of inflammatory T cells and neutrophils. During this process epidermal cells release several cytokines that contribute to the amplification of the immune and inflammatory responses. Anti-inflammatory agents like glucocorticoids, non-steroidal anti-inflammatory drugs can be used with great effectiveness. However, they usually cause adverse effects that limit the possibility of their prolonged use. Growing body of research has been revealing a number of natural products with the ability to reduce most of the damaging effects of solar UVR exposure without causing significant cytotoxicity.

The sunscreens currently available offer some ROS protection mainly through the scattering properties of the sunscreen rather than antioxidant effects (Wang et al., 2011). Addition of antioxidants to sunscreens offers great potential to neutralize the UV-induced free radicals and the idea of combining UV filters with antioxidants is appealing. However, in order to achieve relevant efficacy a number of requirements have to be fulfilled by the end product: (1) a high antioxidant capacity (2) a stable formulation, (3) the ability to penetrate the epidermal barrier and (4) be present at concentrations that cause significant protective effect. Analysis performed in some of the products currently available on the market indicate that further improvements are needed (Wang et al., 2011). The development of adequate formulations that allow the delivery of effective doses of the active ingredient into the epidermis is a major challenge. Nano-formulations, such as nanostructured lipid carriers, liposomes, and nano-emulsions (Hussain et al., 2017) have great potential to resolve the problem of poorly soluble compounds. The development of novel nanoparticles without the use of toxic chemicals, such as silver and gold-nanoparticles will eliminate the risks for their use in cosmetic, pharmaceutical and other biomedical applications (Castangia et al., 2017; Jiménez Pérez et al., 2017). In addition, the combination of two or more natural products with complementary beneficial activities should provide promising new alternatives to be explored. For example, a study revealed that both honokiol and modified citrus pectin induced dose-dependent antioxidant activity in a synergistic manner, inhibiting NF-kB and TNF-α (Ramachandran et al., 2017). The reviewed work suggests that combination of natural products in appropriated formulations represent a viable strategy for the treatment of skin conditions associated with inflammation and oxidative stress.

## Author contributions

SD: Performed literature review and wrote manuscript; LZ: Performed literature review, created figures, assisted in writing manuscript; RO: Assisted in assembling sources, creating bibliography and editing manuscript; LJ: Assisted in assembling sources, creating bibliography and editing manuscript; YZ: Manuscript review; AK: Manuscript review.

### Conflict of interest statement

The authors declare that the research was conducted in the absence of any commercial or financial relationships that could be construed as a potential conflict of interest.
